# Inter-examiner reproducibility of tests for lumbar motor control

**DOI:** 10.1186/1471-2474-12-114

**Published:** 2011-05-25

**Authors:** Flemming Enoch, Per Kjaer, Arne Elkjaer, Lars Remvig, Birgit Juul-Kristensen

**Affiliations:** 1 Department of Rheumatology, Rigshospitalet, Copenhagen University Hospital, Copenhagen, Denmark; 2 Institute of Sports Science and Clinical Biomechanics, University of Southern Denmark, Odense, Denmark; 3 Fredensborg Health Centre, Fredensborg, Denmark; 4 Research Unit for Musculoskeletal Function and Physiotherapy, University of Southern Denmark, Odense, Denmark

## Abstract

**Background:**

Many studies show a relation between reduced lumbar motor control (LMC) and low back pain (LBP). However, test circumstances vary and during test performance, subjects may change position. In other words, the reliability - i.e. reproducibility and validity - of tests for LMC should be based on quantitative data. This has not been considered before. The aim was to analyse the reproducibility of five different quantitative tests for LMC commonly used in daily clinical practice.

**Methods:**

The five tests for LMC were: repositioning (RPS), sitting forward lean (SFL), sitting knee extension (SKE), and bent knee fall out (BKFO), all measured in cm, and leg lowering (LL), measured in mm Hg. A total of 40 subjects (14 males, 26 females) 25 with and 15 without LBP, with a mean age of 46.5 years (SD 14.8), were examined independently and in random order by two examiners on the same day. LBP subjects were recruited from three physiotherapy clinics with a connection to the clinic's gym or back-school. Non-LBP subjects were recruited from the clinic's staff acquaintances, and from patients without LBP.

**Results:**

The means and standard deviations for each of the tests were 0.36 (0.27) cm for RPS, 1.01 (0.62) cm for SFL, 0.40 (0.29) cm for SKE, 1.07 (0.52) cm for BKFO, and 32.9 (7.1) mm Hg for LL. All five tests for LMC had reproducibility with the following ICCs: 0.90 for RPS, 0.96 for SFL, 0.96 for SKE, 0.94 for BKFO, and 0.98 for LL. Bland and Altman plots showed that most of the differences between examiners A and B were less than 0.20 cm.

**Conclusion:**

These five tests for LMC displayed excellent reproducibility. However, the diagnostic accuracy of these tests needs to be addressed in larger cohorts of subjects, establishing values for the normal population. Also cut-points between subjects with and without LBP must be determined, taking into account age, level of activity, degree of impairment and participation in sports. Whether reproducibility of these tests is as good in daily clinical practice when used by untrained examiners also needs to be examined.

## Background

Pain in the lumbar region is a common problem, corresponding to a point prevalence of approximately 15-27% of all adults [1,2]. It is estimated that 60 to 80% of the Danish population will experience low back pain (LBP) sometime during their lifetime [3]. The vast majority of these LBP episodes will settle within two to three months, however more than 70% of those with non-treated LBP will have a recurrence within a year [4,5]. It may be, that problems for the individual patient, are cumulative with each episode of LBP [6,7]. It is disturbing that about 10% of the people having an episode of LBP will develop a chronic pain condition and related disability [8]. Half a year after the first episode of LBP, more than 60% still have pain, and 16% will still be on sick leave [8-10].

There is almost no consensus among different professional groups with regard to examination and treatment methods for patients with low back pain [11]. The lack of a specific diagnosis for the majority of chronic LBP patients has led to the development of many alternative diagnostic assessment processes.

Of increasing interest in recent years has been the assessment of static and dynamic motor control of the lumbo-pelvic complex in LBP, called lumbar motor control (LMC). Various methods of LMC evaluation are currently applied clinically for diagnostic purposes, as part of the physiotherapy examination [5,12-19]. In this study, the evaluation of LMC included tests regarding the ability to control and reposition the lumbo-pelvic complex, when challenged in different directions. The importance of joint stabilisation in its neutral zone has been demonstrated [20,21], and inter-segmental instability and altered recruitment of the stabilising muscles have been proposed as possible contributing factors to the development of LBP [12,13,16,22-24].

An optimum static and dynamic stability of the lumbo-pelvic complex, as an expression of the LMC, is considered important in order to maintain the functional and structural integrity of the lumbar region. Deficits in dynamic stability can compromise segmental spinal stability and may lead to tissue damage, and the development of chronic LBP [24-26]. In particular, the dynamic stability of the lumbo-pelvic complex can be biomechanically challenged by both trunk and limb movements. Appropriate muscle coordination is considered important for the function of the lumbar spine as an effective 'force-bridge' between the trunk, the lower and the upper extremities, as well as for force development within the lumbar region itself. The complex anatomy of the lumbo-pelvic region and the multidirectional functional demands placed on it, constitute a challenge for those responsible for determining a specific structural diagnosis. A diagnosis based on movement control impairment is considered by many authors to be a relevant way to subgroup low back pain patients [13,17,18].

In a number of studies, several tests for LMC and movement control impairment have been evaluated for their reliability [18,19,27-30]. In essence, all these studies report test reproducibility, which ranges from poor to almost perfect, apparently depending on the qualifications of the examiners, the focus of the test (symptoms or alignment/movement), and the number of possible subcategories. Clinically, it is difficult without any technical equipment to visually estimate how much the lumbar region is moving during tests for LMC. Previous studies have judged LMC tests dichotomously, as "can-cannot/yes-no". However, a lot of information is hidden between these two end-points. Besides, there has been no clear consensus for when the test is passed/not passed, or at what level the relevant dichotomous cut-point of each test should be. Consequently, there is a need for more precise test descriptions, in addition to tests with more quantitative and reproducible methods for measuring LMC.

A test battery consisting of five tests, described in several articles and textbooks [12-14,19,31-34], is often used in daily clinical practice. The tests have evolved and been modified over the past ten years, including a method for continuous quantification. This has been done in order to achieve clear standards for quantifying LMC as the tests challenge LMC in three directions: flexion, extension and rotation. Information from these tests contributes to making a directionally specific diagnosis, which should make it possible to design a retraining program and provide more specific advice on appropriate physical activity, including measurement of the effect on LMC. However, the reproducibility of the tests still needs to be determined.

Therefore, the aim of the current study was to test the inter-examiner reproducibility of these tests for LMC in a mixed population of subjects with and without LBP.

## Methods

### Definitions

A reliable test is a test that 'can be depended upon with confident certainty' (that is, it is trustworthy [35]), meaning that it is reproducible, as well as valid. A reproducible test is a test where one can achieve the same result from two or more different measurements. In this study we focus only on test reproducibility.

### Study design

The study was a test-retest reproducibility study with two examiners, who followed a three-phase reproducibility protocol, recommended by the International Academy of Manual/Musculoskeletal Medicine (IAMMM) [30]. Since this study included continuous data, the protocol was adjusted accordingly to a two-phase study, and excluded the overall agreement phase.

In phase one, the five tests (see Table 1), were described in detail by the two examiners A and B (FE and AE). They were both teachers in the Danish Manual Therapy Society, and they had both had 20 years of clinical experience, including experience in using these tests for LMC. Both examiners tested 10 subjects with LBP in an open study, in order to become familiar with the test procedures and the method for interpreting test results, thereby reducing examiner bias.

**Table 1:** Clinical tests for Lumbar Motor Control (LMC)

Test name	Subject position, performance and measurement equipment	Modified by previous test (reference)
**1) Joint position sense (JPS) **	Sitting, feet unsupported, LB in neutral, 5 cm tape-measure, taped at 0 cm at S1, and marked by laser pointer. LB movement from max anterior-max posterior tilt. Subject reposition of LB (neutral), distance measured between 0 cm (S1) and laser pointer.	[13,14]

**2) Sitting Forward lean (SFL) **	Sitting, feet supported, LB in neutral, mark with 15 cm ruler at S1 and 10 cm above. 5 repetitions of hip flexion to max 120°, distance between marks (0 cm and 10 cm) measured (cm).	[13,14,28]

**3) Sitting knee extension (SKE) **	Sitting, feet unsupported, LB in neutral, 5 cm tape-measure, taped at 0 cm at S1, and marked by laser pointer. 5 repetitions in knee extension up to at least -10°, distance measured between 0 cm (S1) and laser pointer.	[31,28]

**4) Bent knee fall out (BKFO) **	Supine lying, one knee flexed 120°, LB and pelvis in neutral. 5 cm tape-measure placed between right and left ASIS, with 0 cm and laser pointer placed lateral to right ASIS. 5 repetitions of abduction/external hip rotation up to max. 45°, distance measured between laser pointer and 0-point (cm).	[17,12-14]

**5) Leg lowering (LL)**	Supine lying, hips flexed 90°, knees maximally flexed, LB in neutral. BPU placed under LB, inflated to 40 mm Hg. LB downward press to increase BPU to 45 mm Hg. 5 repetitions of leg lowering. Increase measured in BPU (mm Hg).	[31,13,14]

LB = low back, ASIS = Anterior Superior Iliac Spine, BPU = Biopressure Unit.

In phase two, the two examiners applied the five tests for LMC on all subjects (40 subjects, 63% of whom had LBP on the day of the examination, see Table 2) in two separate rooms. Each examiner provided the subject with the necessary instruction for the tests, and all subjects were appropriately unrobed to allow visualisation of the lumbosacral spine. The subjects were examined independently and in random order by two examiners on the same day, and after examiner A had tested a subject, the subject was examined by examiner B, and vice versa. Half the subjects started with examiner A, and half with examiner B. Both examiners performed the tests in the same order on each subject, specified in the current manuscript (Test 1-5) in the section 'Tests for lumbar motor control'.

**Table 2:** Demographics obtained by questionnaires and Numeric Pain Rating Scale (NPRS)

Variable	People with LBP on day of examination (n = 25 (62.5% of total))	People without LBP on day of examination (n = 15 (37.5% of total))
Age in years (mean (SD)	47 (12)	45 (19)
Gender (n (Male/Female)	11/14	3/12
(% (Male/Female))	(44/56)	(20/80)
Previous episodes ever (n (% of group))		
None	2 (8)	9 (60)
One	1 (4)	1 (7)
Less than 5	4 (16)	2 (13)
5 or more	18 (72)	2 (13)
More than 10		1 (7)
Pain on day of examination * (n (% of group))		
No pain		15 (100)
1-3	13 (52)	
>3	12 (48)	
Back history in months (n (% of group))		
0-3	2 (8)	12 (80)
4-7	2 (8)	2 (13)
8-12	13 (52)	1 (7)
13-16	8 (32)	

LBP = Low Back Pain.

* Numeric Pain Rating Scale (NPRS range 0-10)

Healthy controls were included in order to maximise variability in the subjects' test performance, partly to reduce examiner bias and partly to cover the spectrum of all possible measurement levels available for the tests.

The Regional Committee on Biomedical and Research Ethics approved the study (H-A-2008-082), which includes the principles of the Declaration of Helsinki. All participants gave their consent after receiving oral as well as written information about the study.

### Study sample

The LBP subjects were recruited from patients seeking care from three different private physiotherapy clinics. The non-LBP subjects were recruited from the clinical staff's aquaintances, as well as from patients without back pain problems.

The inclusion criteria were men and women, aged 18-85 years, with (25 subjects) or without (15 subjects) non-specific LBP problems (see Table 2), while the exclusion criteria were neurological or rheumatologic disorders, acute pain in the hip and leg, diabetes and cancer, and inability to speak and understand Danish. The Numeric Pain Rating Scale (NPRS), previously shown to be valid [36], was used to describe the severity of the LBP.

### Tests for lumbar motor control (LMC)

Five different tests for LMC were used, including one for repositioning (RPS) and four for dynamic stability, including sitting forward lean (SFL), sitting knee extension (SKE), bent knee fall out (BKFO) and leg lowering (LL) (Table 1). Generally, the subjects performed a maximum of 10 repetitions of each test. The subjects were allowed to practice the RPS test twice, and the remaining four dynamic stability tests a maximum of five times were allowed, before the test examination started. Thereafter, three repetitions of the RPS test and five repetitions of the other tests were performed, and the mean value of these was calculated. Within this range, the amount of instruction and tactile feedback before the test evaluation started varied among subjects, depending on the subject's ability to understand and perform the tests. The tests are summarised in Table 1.

*1) Repositioning (RPS) *was performed by measuring how accurately the subject during sitting could re-position the low back (LB) into the former lumbar position, after having actively moved around, in flexion and extension. The subject was sitting with feet supported, and the examiner guided the subject's LB into neutral position. The examiner ensured that the LB was in neutral position, i.e. midway between the posterior and anterior tilt. A 5 cm tape-measure with mm markings was placed on the LB with the 0 cm marking on Sacral segment 1 (S1), as the caudal end of the tape measure. A laser pointer (Class 3A Laser product, Wen Zhou Xinke, China), placed on a stable base and adjusted to be level, was positioned to have the mark line directly on 0 cm. The subject was instructed to remember this position, and then to move the pelvis twice from the maximum anterior to the maximum posterior tilt and then return to the neutral position. With the laser line on the tape-measure, the deviation from the 0 point was measured in cm, and this could be read within 0.25 mm accuracy. The test was performed three times.

*2) Sitting Forward Lean (SFL) *was designed to measure the amount of LB movement that was necessary for a sitting forward leaning movement of the upper body. The range of motion (ROM) of the LB was measured by a 15 cm ruler. The subject was sitting upright with the knees and the hips at 90°, and with the hands resting on the thighs. The examiner placed the subject's LB in neutral position and marked the SI point and a point 10 cm cranially, using a pen on the skin. The subject was instructed to hold that position of the two points relative to each other, during the subsequent movements. To guide the range of movement, the examiner firmly grasped the subject's pelvis and moved the pelvis anteriorly, until a maximum of 120° hip flexion was reached, measured by a plurimeter V gravity inclinometer (Access Health, Melbourne, Australia), placed on the LB. The examiner placed the LB in neutral position, i.e. midway between the posterior and anterior tilt. The subject was then instructed to remain in neutral position of the LB, while moving the trunk and pelvis forward until the hips reached 120° flexion or within the available ROM. Initially, the first test performance movement was guided with tactile feedback, by the examiner's 1st and 2nd finger on the S1 and 10 cm mark on the tape-measure. Once the subject was well instructed, the examination of the test started, and the subject performed five repetitions without feedback. At the forward lean position of each repetition, the distance between S1 and 10 cm mark was measured in cm with a ruler, to within one decimal point.

*3) Sitting knee extension (SKE) *was designed to determine the magnitude of LB movements that occurred during a sitting knee extension, using a tape-measure (in cm). Using the same setup as with RPS, the couch was raised until the subject's feet were off the floor. In order to define ROM at the knee during the test, the examiner manually fixed the subject's pelvis in neutral with one hand, and extended the knees as much as possible, however only to a maximum of minus 10° extension. This was controlled using the plurimeter, placed at the tibia just distal to the tibial tuberosity. A 5 cm tape-measure was placed on the LB with 0 cm at S1, and with the laser pointing at 0 cm as the caudal end of the tape measure. The examiner ensured that the LB was in neutral position, i.e. midway between the posterior and anterior tilt. The subject was instructed to remain in a neutral position of the LB, while moving the knee to minus 10° extension or within the available ROM. Initially, the movement was guided with feedback by the examiner's 1st and 2nd finger placed on the S1 and 10 cm mark, previously marked on the skin cranially to the S1. Once the subject was well instructed, the test started, and the subject performed five repetitions without feedback. At the end of each knee extension, the LB movement was measured as the distance from 0 cm to the laser pointer mark.

*4) Bent Knee Fall Out (BKFO) *was designed to evaluate the range of LB movement that takes place during a supine lying external rotation of the hip, using a tape-measure (in cm). The subject was supine lying with right hip flexed, the knee flexed at 120°, with the feet resting on the surface of the couch, and the arms lying relaxed beside the body. The examiner ensured that the LB was in neutral position, i.e. midway between the posterior and anterior tilt. A 5 cm tape-measure was placed laterally to the anterior superior iliac spine (ASIS opposite to the bent leg) with 0 cm laterally placed on the ASIS and pointing laterally towards the laser. The laser line was adjusted to the 0 cm point to determine the amount of lateral hip movement, pointing medially to the 0 cm mark on the tape-measure. The examiner manually fixed the subject's pelvis and moved the hip of the bent leg into as much abduction/external rotation as possible, however, only to a maximum of 45°, measured by the plurimeter, placed at the medial side of the knee. The subject was instructed to abduct the knee to the determined point and return to the starting position, and in the beginning, the subject received feedback via the examiner's finger on the ASIS in order to detect the movement. Once the subject was well instructed, the test started, and the subject performed five repetitions without feedback. At the extreme of hip abduction in each repetition, the LB movement was measured as the distance from the 0 cm on the tape-measure to the laser pointer mark.

*5) Leg Lowering (LL) *was designed to quantify the extent of LB movement accompanying a supine lying unilateral leg lowering using a pressure biofeedback unit (PBU) (Chattanooga Ltd Hixson, USA). The PBU instrument was developed to monitor LMC by recording pressure changes in mm Hg during the five repetitions. The PBU has been shown to be reliable and capable of detecting even small changes in pressure during movement [37]. The subject was placed in supine position with the hips at 90° flexion. The knees were in maximum relaxed flexion. The examiner ensured that the LB was in neutral position, i.e. midway between the posterior and anterior tilt, and the ASIS were at a horizontal level. The arms were relaxed and beside the body. A BPU was placed under the LB, and inflated to 40 mm Hg. First, the subject was asked to actively push the LB downwards, increasing the BPU pressure to 45 mm Hg. Then the subject was instructed to lower the feet to just above the surface of the couch. In the early attempts, the subject was allowed to have visual feedback from the BPU. Once the subject was well instructed, five repetitions were performed without feedback. At each repetition, the pressure in mm Hg was recorded, when the feet were as close as possible to the couch.

### Statistical analyses

**Figure 1:**
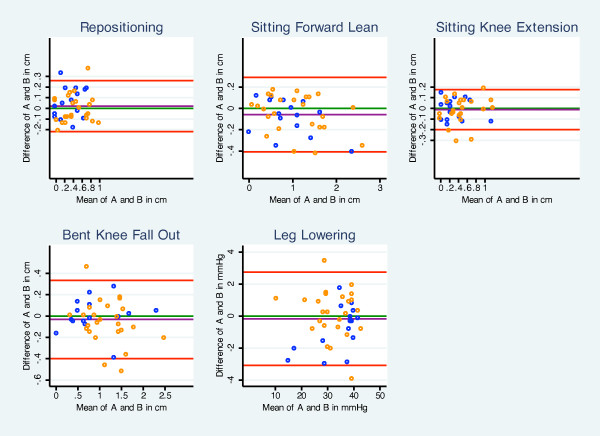
**Bland and Altman plots (differences between examiner A and B measures (y-axis), and the mean of examiner A and B (x-axis) for each of the tests), with 95% limits of agreement (LOA) for the five tests (red lines)**. The green line (y = 0) is perfect average difference, and the purple line is the observed average difference (n = 38 for Sitting Knee Extension, n = 40 for the remaining tests; Leg Lowering in mm Hg, remaining tests in cm). Examiner A and B represent the examiners named A and B. Blue dots represent subjects without Low Back Pain (LBP), while red dots represent subjects with LBP on day of examination.

For each of the tests, the total mean is reported together with the standard deviation. To evaluate the inter-examiner reproducibility of test performance, intraclass correlation coefficients (ICC) type 2.1 [38,39] and Bland and Altman's [40] limits of agreement (LOA) were used (Figure 1). In order to give clinicians information about the minimal change that is not due to error, the minimal detectable change (MDC) was calculated for each test.

A correlation coefficient above 0.90 is considered 'excellent' reproducibility, greater than 0.75 is considered 'good' reproducibility, and less than 0.75 indicates 'poor' reproducibility [41]. The ICC was calculated for all subjects as a group, and separately for those with and without LBP on the day of the examination.

LOA is based on the difference between results from examiners A and B. The average of the differences in measures from examiner A and B is reported (Table 3), together with the standard deviation and the range within 95% of the differences (95% LOA). Data are presented for the groups with and without LBP on the day of the examination, separately, in addition to the whole group.

**Table 3:** Summary statistics (mean, SD, range) are given for examiner A, examiner B and both examiners A + B together, on each of the five tests for lumbar motor control

Variable	N	Mean (SD)	Range
*Examiner A*			
1) Repositioning, RPS (cm)	40	0.37 (0.30)	(0.00-1.00)
2) Sitting Forward Lean, SFL (cm)	40	0.98 (0.59)	(0.04-2.50)
3) Sitting Knee Extension, SKE (cm)	38	0.39 (0.29)	(0.00-1.05)
4) Bent Knee Fall Out, BKFO (cm)	40	1.05 (0.50)	(0.00-2.40)
5) Leg Lowering, LL (mm Hg)	40	32.80 (7.23)	(12.00-43.40)

*Examiner B*			
1) Repositioning, RPS (cm)	40	0.35 (0.27)	(0.00-0.92)
2) Sitting Forward Lean, SFL (cm)	40	1.04 (0.65)	(0.12-2.90)
3) Sitting Knee Extension, SKE (cm)	38	0.40 (0.30)	(0.05-1.00)
4) Bent Knee Fall Out, BKFO (cm)	40	1.09 (0.55)	(0.10-2.55)
5) Leg Lowering, LL (mm Hg)	40	32.97 (7.06)	(11.20-42.40)

*Examiner A + B*			
1) Repositioning, RPS (cm)	80	0.36 (0.27)	(0.00-1.00)
2) Sitting Forward Lean, SFL (cm)	80	1.01 (0.62)	(0.04-2.90)
3) Sitting Knee Extension, SKE (cm)	76	0.40 (0.29)	(0.00-1.05)
4) Bent Knee Fall Out, BKFO (cm)	80	1.07 (0.52)	(0.00-2.55)
5) Leg Lowering, LL (mm Hg)	80	32.89 (7.10)	(11.20-43.40)

Bland and Altman plots were constructed by plotting the differences between A and B measures (y-axis) against the mean of A and B (x-axis) for each of the tests, as shown in Figure 1. The green line (y = 0) is perfect average difference and the purple line is the observed average difference. The distance between these lines indicates the bias towards one of the observers' measures. The distance from the purple line (average difference) to each dot represents the difference between the examiners rating corresponding to the observed mean value on the x-axis for the two examiners. The red lines in the figures indicate 95% LOA as described above. The closer the dots are to the green line within LOA, the less disagreement in measures. People with and without LBP on the day of the examination are marked with orange and blue symbols, respectively.

The standard error of measurement (SEM) was calculated, as suggested by de Vet et al, 2006, using the formula: SEM = Standard deviation of the mean differences between tester A and B divided by √2 [42]. Thereafter the minimal detectable change (MDC) was calculated using the formula: MDC = SEM × √2 × 1.96 [43].

For statistical analyses, the STATA statistical package was used (Stata Corp., 2000, Stata Statistical Software: Release 11.1, College Station, TX). The command "icc23" (two way ANOVA) was used to calculate ICC type 2.1 with 95% confidence intervals (CI), and the command "concord" was used to calculate LOA, as well as Bland and Altman plots.

## Results

In total, 40 subjects were recruited for this reproducibility study, of whom 14 were men and 26 were women, having an age range from 20 to 82 years. The mean age of the subjects was 46.5 years (SD 14.8) (Table 2), and 15 (37.5%) of them did not have pain on the test day. Pain intensity, measured by the NPRS on the test day, ranged from 0 to 8. In total, nine subjects (22.5%) had never had backache for more than three days. In contrast, 19 subjects (47.5%) had had more than 10 episodes of LBP, lasting more than three days.

Summary statistics are given for the two examiners (A, B) on each of the five tests for LMC (Table 3). All of the tests (SFL, SKE, BKFO, LL, and RPS) had excellent inter-examiner reproducibility (ICC >0.93) for the whole group (Table 4), with MDCs between 0.19 cm and 0.37 cm for four of the tests, and 2.90 mm Hg for the LL (Table 4). The Bland and Altman plots showed that the majority of the differences were less than 0.2 cm for the whole group (Figure 1). From the LOA 95% of the measurements' variation is within the range of -0.44 cm to 0.35 cm for tests 1-4, while for test 5 (LL) the range is from -3 to 3 mm Hg, representing the absolute measurement differences in relation to the mean of the measurements. In the Bland and Altman plots, most of the measurements are located within a smaller range (Figure 1). When analysing groups with and without LBP on the day of the examination separately, more subjects with LBP had values in the outer range of LOA, but the ICC values were about the same level as for the whole group.

**Table 4:** Minimal Detectable Change (MDC), Intraclass Correlation Coefficient (ICC type 2.1), with 95% confidence intervals (95% CI) for the five lumbar motor control tests

Test	MDC	ICC	(95% CI)	Difference Average (SD)	95% LOA
1) Repositioning, RPS *(all)*	0.24	0.90	(0.81; 0.94)	0.02 (0.12)	(-0.22; 0.26)
-> LBP = 0	0.85	(0.60; 0.95)	0.06 (0.13)	(-0.19; 0.31)	
-> LBP = 1	0.92	(0.82; 0.96)	0.00 (0.12)	(-0.23; 0.22)	
2) Sitting Forward Lean, SFL *(all)*	0.35	0.96	(0.92; 0.98)	-0.06 (0.18)	(-0.41; 0.29)
-> LBP = 0	0.94	(0.82; 0.98)	-0.08 (0.18)	(-0.43; 0.28)	
-> LBP = 1	0.96	(0.92; 0.98)	-0.05 (0.18)	(-0.40; 0.30)	
3) Sitting Knee Extension, SKE *(all)*	0.19	0.95	(0. 90; 0.97)	-0.01 (0.10)	(-0.20; 0.18)
-> LBP = 0	0.94	(0.84; 0.98)	0.00 (0.08)	(-0.17; 0.16)	
-> LBP = 1	0.95	(0.88; 0.98)	-0.02 (0.11)	(-0.22; 0.19)	
4) Bent Knee Fall Out, BKFO *(all)*	0.37	0.94	(0.88; 0.97)	-0.03 (0.19)	(-0.40; 0.34)
-> LBP = 0	0.97	(0.92; 0.99)	0.01 (0.15)	(-0.29; 0.31)	
-> LBP = 1	0.89	(0.77; 0.95)	-0.06 (0.21)	(-0.46; 0.35)	
5) Leg Lowering, LL *(all)*	2.90	0.98	(0.96; 0.99)	-0.17 (1.49)	(-3.08; 2.74)
-> LBP = 0	0.98	(0.92; 0.99)	-0.75 (1.43)	(-3.54; 2.05)	
-> LBP = 1	0.98	(0.96; 0.99)	0.18 (1.44)	(-2.65; 3.00)	

Average refers to the mean difference between the two examiners A and B in cm (for Leg Lowering in mm Hg) with standard deviation (SD). The 95% Limits Of Agreement (95% LOA) describes the interval in which 95% of the differences between examiner A and B are located. Values are given for all, and for the subsamples without (LBP = 0) and with Low Back Pain (LBP = 1) on day of examination.

## Discussion

The principal findings were a good to excellent inter-examiner reproducibility of the five tests for LMC, with the ICC ranging from 0.90 to 0.98 for the whole group, with a difference between the two examiners of less than 0.2 cm, and a low MDC (0.19-0.37 cm¸2.90 mm Hg). To our knowledge, this is the first study reporting excellent reproducibility for tests of LMC, using a quantitative method.

Previously, all five studies on reproducibility of LMC tests have been studied in a dichotomous setup with qualitative data. Three studies have shown substantial and almost perfect inter-tester reproducibility in qualitative ratings of similar LMC tests [18,19,27]. In two of the studies, "reproducibility of specific classification systems for motor control impairments was tested", in which kappa was 0.96 for experienced and 0.61 for inexperienced clinicians [18], respectively 0.75 for experienced clinicians [27]. However, both these studies analysed the diagnostic reproducibility based on a whole battery of tests, and thus they are not comparable with each individual test in the current study. The third study, which tested reproducibility of individual tests for LMC, rated dichotomously, showed kappa values of 0.72 for SKE, and 0.38 for BKFO [19], both of which were not as reproducible as in the current study (0.95 and 0.94). Further, only six out of the ten tests for LMC were classified as having substantial reproducibility with kappa > 0.60 [19]. Of these ten tests, only SKE and BKFO are comparable with the current tests (same position, same test procedure, rating deficits in same direction, although dichotomously). A similar study of LMC tests showed substantial kappa values for hip extension with 0.72 and 0.76 (left and right) for 80% of the cases, but neither test was included in the current study [29].

Inter-examiner reproducibility of 53 different tests showed kappa to be ≥0.75 for tests related to symptoms, but when related to alignment and movement, kappa was only ≥0.41[28]. Two of these tests (SKE, BKFO) were similar to the current tests with kappa of 0.58 and 0.52; however, test results in that study were rated dichotomously (yes/no), and solely rated by visual observations. Since it is well known that 'judgments based on visual and tactile information are often difficult to make reliable' [28], use of a visual rating method may have been one of the reasons for the poor kappa values.

However, in kappa studies it is essential to secure a high overall agreement, and a 50/50% prevalence of positive and negative findings in order to measure the true reproducibility of the test (34). We are not provided with this information in the above-mentioned papers and consequently, the true kappa value may be higher than presented by the authors. The strengths of this study are, that despite the differences in study design, tests, examiner expertise, and selection criteria for the study population, the results are in line with data from previous studies and show better reproducibility. This may be due to our use of the standardised protocol by IAMMM [30], including a standardised training procedure for the examiners, a protocol with well defined procedures and operational definitions that provided quantifiable values.

Also, in this protocol, the two examiners went through a training phase in order to minimise bias during performance of the tests and to increase their overall agreement on test performance and interpretation. This precaution will increase the intention of the study to test the test independent of the examiner, and not to test the combination of test and examiner, illustrating that the tests *per se *are good to excellent. Finally, the study was carried out on both LBP and non-LBP subjects, for whom the test battery is intended, making the results relevant for screening purposes within this group.

The weakness of the study is that we do not know the reproducibility of the current tests carried out by inexperienced clinicians, which of course might be different from the reproducibility of experienced and trained examiners, as also shown in previous studies [18,19]. However, the current tests were developed to include only quantifiable variables, and do not include other more subjective factors, such as breathing, co-contraction, rigidity and perceived effort, which are commonly included in daily clinical practice. This absence is likely to have increased the observed reproducibility, but reproducibility also needs to be tested in a more normal clinical environment.

Further, in case inexperienced examiners have a low inter-examiner test reproducibility, our study has shown that it should be possible through education and training to obtain high enough skills to perform the tests in a reproducible way.

Another reason for the high reproducibility may be that day to day variability is not tested in the present reproducibility study, since the subjects were examined twice within the same hour. For that reason, the day-to-day variation needs to be tested in a future study.

The use of correlation coefficients for reliability can easily disguise large differences in measurements. Therefore, also the Bland and Altman plots [40] were used, from which the variation in each measure from each examiner is demonstrated. This provides the reader with the true variation, as a supplement to the ICC. Further, MDC is provided for clinical practice, to give an idea of how much change in LMC over time is needed, to exceed the measurement error.

Several aspects need to be considered and analysed in the future: Since an excellent reproducibility of clinical tests for LMC was obtained, the relevant cut-point (distance moved from the 0 point) for abnormality for each of the tests must be determined by testing the human variation in the normal population. Further, the validity must be tested, i.e. the discriminative ability of the tests to discriminate between subjects with and without LPB in a larger study sample.

A recent pilot study showed the predictive validity of a poor performance on two selected LMC tests in relation to an increased risk of lower limb/lumbar spine injuries in professional dancers [12]. Another pilot study (without a control group) including 38 LBP patients showed that after treatment focusing specifically on increasing LMC, the pain decreased, and, physical function and LMC improved [33]. The addition of tests for LMC with excellent reproducibility may also enhance future validity studies, such as those previously described in other positions [12] and other movement directions [29]. Using a whole test battery may make it possible to determine the optimum number and combination of tests with the highest diagnostic accuracy (i.e. sensitivity and specificity). The future of LMC tests is challenging, and further studies of these interactions are required.

## Conclusion

The current five tests for LMC had excellent (RPS, SFL, SKE, BKFO and LL) reproducibility. However, reproducibility is only the first step on the path to establishing the diagnostic value of these tests. Therefore, subsequent studies need to include larger cohorts of subjects, including establishment of values for the normal population, and cut-points between subjects with and without LBP, while taking into account age, levels of activity, degree of impairment and participation in sports. Further, establishment of the reproducibility of these tests in normal clinical practice must be performed.

## Competing interests

The authors declare that they have no competing interests.

## Authors' contributions

FL was involved in the planning and acquisition of the data, the making of the videos, the data analysis and the writing of the paper. AE was involved in the planning and acquisition of the data, LR and BJK were involved in the planning, methodological considerations, analysis of the data, and revision of the paper. PK was involved in the data analysis, calculation of the statistics and revision of the paper. All authors read and approved the final manuscript.

## Pre-publication history

The pre-publication history for this paper can be accessed here:

http://www.biomedcentral.com/1471-2474/12/114/prepub
